# A Manual of Medical Jurisprudence and Toxicology

**Published:** 1896-08

**Authors:** 


A Manual of Medical Jurisprudence and Toxicology. By Henry C. Chapman, M. D., Professor of the Institutes of Medicine and Medical Jurisprudence in Jefferson Medical College of Philadelphia; Member of the College of Physicians of Philadelphia, etc., etc. Second edition, revised. With fifty-five illustrations and three plates in colors. Saunders' New Aid Series. Small 8vo, pp. 254. Price, $1.50. Philadelphia: W. B. Saunders. 1896.

The first edition of this work was reviewed in the December, 1893, number of the Journal, page 314, and the criticisms there noted have in a measure been acted upon. The text and scope of the new edition is practically the same as in the former edition, but has been greatly improved by the addition of many illustrations and tables. Bibliographical notices have also been inserted and the volume now is a welcome addition to the most interesting of all the medical sciences. W. C. K.

